# Phytochemical screening and in-vitro biological properties of unprocessed and household processed fenugreek (*Trigonella foenum-graecum* Linn.) seeds and leaves

**DOI:** 10.1038/s41598-023-31888-y

**Published:** 2023-04-29

**Authors:** Shaimaa G. Abdel Salam, Mohamed M. Rashed, Nabih A. Ibrahim, Emam A. Abdel Rahim, Tahany A. A. Aly, Ammar AL-Farga

**Affiliations:** 1 Food Technology Research Institute, Agricultural Research Center, Giza, 12613 Egypt; 2 Biochemistry Department, Faculty of Agriculture, Cairo University, Giza, 12613 Egypt; 3 Regional Centre for Food and Feed, Agriculture Research Center, Ministry of Agriculture, Giza, Egypt; 4 Department of Biochemistry, College of Sciences, University of Jeddah, P.O. Box 34, Jeddah, 21959 Saudi Arabia

**Keywords:** Biochemistry, Biological techniques

## Abstract

The impact of household processes on fenugreek leaves and seeds has been analyzed for total phenolic (TP) and total flavonoid content (TF), and in-vitro biological activities such as antioxidant, antimicrobial, and anti-inflammatory properties. Processes included air-drying for leaves and germinating, soaking, and boiling for seeds. Air-dried fenugreek leaves (ADFL) had high TP (15.27 mg GAE g^−1^ D.W.) and TF (7.71 mg QE g^−1^ D.W.) (milligram quercetin equivalents per gram dry weight). The TP contents of unprocessed, germinated, soaked, and boiled seeds were 6.54, 5.60, 4.59, and 3.84 mg gallic acid equivalents per gram of dry weight (mg GAE g^−1^ D.W.), respectively. The TF contents in unprocessed fenugreek seeds, germinated fenugreek seeds, soaked fenugreek seeds, and boiled fenugreek seeds (BFS) were 4.23, 2.11, 2.10, and 2.33 mg QE g^−1^ D.W., respectively. Sixteen phenolic and nineteen flavonoid compounds has been identified using high-performance liquid chromatography. Antioxidant activity using 2,2-diphenyl-1-picrylhydrazil (DPPH^·^), 2,2-azinobis (3-ethylbenothiazoline-6-sulfonic acid (ABTS^+·^), and ferric reducing antioxidant power (FRAP^·^) assays indicated that ADFL had the highest activity. Antimicrobial activity has been evaluated against each of the eight pathogenic bacterial and fungal strains. ADFL showed the strongest activity with minimum inhibitory concentrations values ranging from 0.03 to 1.06 and 0.04 to 1.18 mg ml^·1^ against bacterial and fungal strains, respectively. Anti-inflammatory activity was evaluated in-vitro against RAW 264.7 macrophage cells using the nitric oxide (NO) assay. Results revealed that ADFL had the highest cytotoxicity and anti-inflammatory activity according to the NO assay. Household processes significantly reduced the in-vitro biological properties of processed seeds.

## Introduction

Phytochemical screening is a method of investigating the existence or absence of potential phytochemical components in the plant. This assay can help scientists to diagnose the bioactive components in plants. It advises not only on the existence of therapeutic agents but also gives information about the presence of economically active ingredients, such as vitamins, phenolics, alkaloids, saponins, tannins, oils, gums, and precursors for the synthesis of complex compounds, etc. Phenolic constituents in plants have potential health activities principally due to their antioxidant activities, such as metal chelation, electrophile scavenging, and reactive oxygen species (ROS) scavenging and inhibition^1^.

Fenugreek (*Trigonella foenum-graecum*) is an annual plant belonging to the family *Fabaceae*. The green leaves and seeds of fenugreek are widely used in food and medicinal applications dating back to ancient times. However, the seeds are sour in taste due to the presence of bitter saponins, which limit their acceptability in foods^2^. It has been possible to decrease the bitter taste of fenugreek seeds by using diverse household processes, such as soaking, germination, boiling, fermentation, etc.^2,3^. Many extracts of each seed or leaf and its active components have been studied for their pharmacological effects and have been reported to have hypocholestrolaemic, antidiabetic, anti-inflammatory, antiulcer, analgesic, antipyretic, CNS-stimulant, antioxidant, wound healing and immune modulatory activity as well as gastro-protective and chemo-preventive activities^4^.

Newly, various actions and behaviours of macrophage cells play an important role in surviving homeostasis and normal physiological conditions by manufacturing a diverse range of biological impacts^5^. Macrophages (RAW 264.7) have been chosen because they are accepted as targeted single cells for assessing immune reactivity^6^. Macrophages can be induced by lipopolysaccharide (LPS) to generate pro-inflammation molecules (e.g., NO and PGE2) through enhancing intracellular signaling pathways including NF-κB and MAPK^7^.

Nitric oxide (NO) is a signaling molecule that plays a crucial role in the way inflammation develops. It has anti-inflammatory activity in normal physiological situations, but it is regarded as a pro-inflammatory mediator in unusual conditions due to overproduction. NO has been generated and released to the endothelial cells. It is an active neurotransmitter at the neuronal synapses, and it also helps to regulate programmed cell death regulation. NO participates in the way the inflammatory disorder in joints, intestines, and in the upper and lower respiratory systems. Hence, NO inhibitors represent significant therapeutic advances in the control of inflammatory diseases^8^. It also has an important property as a bio-regulation molecule in the nervous, immune, and cardiovascular systems. The permanent release of NO is associated with several diseases, including; inflammation, cancers, and arthritis^9^.

RAW 264.7 macrophages induced by LPS are used to assess the in-vitro potential inhibitory effects of anti-inflammatory compounds. LPS is considered one of the most important stimuli used to upgrade pro-inflammatory protein arranging, such as inducible nitric oxide synthase (i–NOS) and cyclooxygenase-2 (COX-2), which are in charge of the high standards of prostaglandin spotted in various inflammatory confuses. Furthermore, i-NOS develop huge quantities of NO, which is considered to be important for showing a necessary role in the inflammation^10^. Beside, the highly reactive nitric oxide becomes more sensitive when it is linked with oxygen and thus produces highly reactive compounds which can cause some provisions cellular damage such as; cellular DNA fragmentation and lipid peroxidation^11^.

This work aimed to investigate the influences of household treatments, including air-drying for fresh fenugreek leaves and each soaking, germination, and boiling for edible fenugreek seeds, on both phenolic, flavonoid and isoflavonoid components, as well as on their in-vitro antioxidant, antimicrobial and anti-inflammatory activities of the selected samples.

## Results and discussion

### Total phenolic (TP) and total flavonoid (TF) content

Extraction with alcohol of 80% for all studied samples produced the following contents of crude extracts, 23.66% for ADFL, 22.64 for UFS, 19.08% for SFS, 20.15% for GFS and 18.91% for BFS.

**Figure 1:**
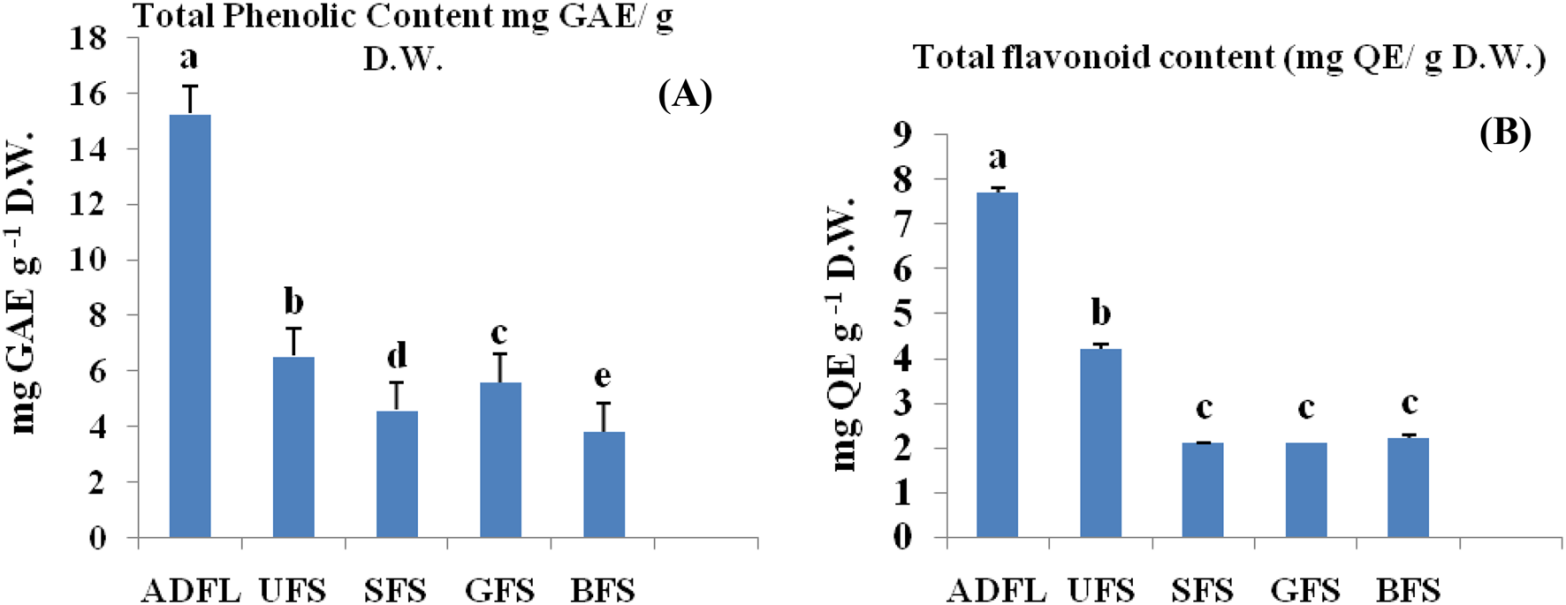
(**A**) Total phenolic content (TP), (**B**) Total flavonoid content (TF) of crude extracts of air-dried fenugreek leaves (ADFL), unprocessed fenugreek seeds (UFS), soaked fenugreek seeds (SFS), germinated fenugreek seeds (GFS) and boiled fenugreek seeds (BFS).

The values of total phenolic content (TP) and flavonoid content (TF) of alcoholic extracts of fenugreek leaves and household processed seeds have shown in Fig. 1. Among all studied samples, ADFL exhibited the highest significant value of TP (15.27 mg GAE g^−1^ D.W.) and TF (7.71 mg QE g^−1^ D.W). Hussain et al.^12^ found that TP and TF in dried fenugreek leaves were 425.4 and 205.1 mg 100 g^−1^, respectively.

On the other hand, UFS had 6.54 mg GAEg^−1^ D.W. of TP and 4.23 mg QE g^−1^ D.W of TF. These amounts were significantly decreased after the household treatments as the following; soaked, germinated, and boiled fenugreek seeds contained 4.59, 5.60, and 3.84 mg GAEg^−1^ D.W. for TP and, 2.11, 2.10 and 2.33 mg QE g^−1^ D.W, respectively, for TF.

These results are in agreement with the earlier findings observed by Hooda and Jood^13^ and Shakuntala et al.^14^ who found that polyphenolic content and antioxidant activity declined after germination treatment. In contrast, another trend, an increase in TP after germination, had been investigated by Pająk et al.^15,16^. Pandey and Awasthi^17^ also approved that soaking and germination enhanced total phenolic content and the antioxidant activity of fenugreek seed flour as compared to raw fenugreek seed flour.

### The profile of phenolic, flavonoid and isoflavone components

Vegetables and cereals (including seed and sprouts) are excellent sources of phenolic components. Legumes contain a high concentration of isoflavonoids^18^.

The phytochemical screening of each phenolic, flavonoid and isoflavonoid component evaluated using of HPLC has shown in the Table 1.The highest concentrations have obtained by the alcoholic crude extract of ADFL, UFS and GFS.

**Table 1:** Phenolic, flavonoids and isoflavonoids screening of crude alcoholic extracts of air-dried fenugreek leaves (ADFL), unprocessed fenugreek seeds (UFS), soaked fenugreek seeds (SFS), germinated fenugreek seeds (GFS) and boiled fenugreek seeds (BFS) by HPLC (mg 100 g^−1^ D.W.).

Compound	Rt* (min)	ADFL	Seeds			
			UFS	SFS	GFS	BFS
Phenolic compounds						
Pyrogallol	4.823	55.83	48.11	51.71	46.25	69.88
Gallic acid	5.347	0.73	0.42	0.66	1.13	0.29
3-hydroxy tyrosol	6.227	5.39	0.42	0.40	1.56	0.20
Catechol	6.727	1.91	0.29	0.23	0.50	0.05
4-amino benzoic acid	6.801	5.59	0.23	0.08	1.21	0.09
Chlorogenic acid	7.701	81.78	5.26	7.84	7.22	3.82
Benzoic acid	7.767	10.56	2.51	3.78	3.17	1.83
*P*-hydroxy benzoic	7.770	14.69	3.50	5.26	4.41	2.54
Vanillic acid	8.303	4.80	18.26	21.40	6.84	6.95
Caffeic acid	8.372	1.64	4.01	3.83	4.14	1.47
Caffeine	8.482	6.46	32.86	40.85	66.34	12.32
Ferulic acid	10.026	20.07	7.48	8.26	18.91	5.27
Salicylic acid	10.917	14.99	5.04	5.31	14.85	0.00
Oleoropine	11.195	162.73	20.68	0.00	50.88	5.16
Ellagic acid	11.534	23.60	2.67	51.71	6.12	1.14
Coumarin	11.779	11.28	0.89	0.66	1.18	0.84
Flavonoid compounds						
Isorhamnetin	2.352	1.57	1.25	1.76	2.69	1.56
Catechin	7.122	77.65	14.19	12.36	40.09	4.15
Apigenin-6-*O*-arabinose-8-*O*-galactose	10.040	71.19	38.65	40.20	80.55	28.17
Hesperidin	10.753	24.60	83.70	67.45	110.30	102.00
Luteoline-7-*O*-glucoside	10.795	2.73	2.34	1.34	2.55	2.40
Rosmarinic	10.825	4.28	1.01	0.64	2.20	1.57
Rutin	10.971	1.63	3.30	6.32	10.75	3.32
Apigenin-7-*O*-glucoside	11.527	20.06	1.59	1.95	3.64	0.55
Quercitrin	11.909	4.92	15.93	11.32	12.29	27.65
Naringin	13.096	1.77	0.86	1.05	1.61	0.20
Naringenin	13.112	5.26	0.32	0.25	0.44	0.75
Quercetin	13.253	9.84	1.58	1.48	2.39	0.71
Kaempferol-3,2-*p*-coumaroyl glucose	13.809	48.04	3.77	4.69	8.57	1.00
Kaempferol	14.599	3.15	0.51	0.36	0.84	ND
Apigenin	14.886	3.21	0.35	0.03	0.08	0.09
Isoflavonoids(Phytoestrogens)						
Daidazein	3.102	3.14	0.48	0.13	0.79	0.14
Genistein	3.934	0.85	0.21	0.39	0.69	0.10
Isoformononetin	6.153	3.87	0.10	0.20	0.26	0.05
Biochanin A	9.272	0.46	0.01	0.02	0.03	0.01

*Rt* retention time, *ND* not detected.

The contents (mg 100 g^−1^ D.W.) of phenolic compounds in ADFL were declined in the following order; oleoropin > chlorogenic acid > pyrogallol > ellagic acid > ferulic acid > salicylic acid > *p*-hydroxy benzoic acid > coumarin > benzoic > caffeine > 4-amino benzoic > 3-OH tyrosol > vanillic > catechol > caffeic. This finding is compatible with Hussain et al.^12^ who found that, among the hydroxyl benzoic acid derivatives, the most abundant acids present in fenugreek leaves were *m* and *p*-hydroxy benzoic acid, β-resorcylic acid, gentisic acid, and gallic acid. In the case of hydroxyl cinnamic acid derivatives, the major acids present included; *o* and *m*-coumaric acid and sinapic acid. HPLC analysis showed the absence of α-resorcylic acid, vanillic acid, and *p*-coumaric acid in air-dried fenugreek leaves.

The dominant components in UFS were pyrogallol, caffeine, oleoropin, vanillic, ferulic, chlorogenic, salicylic, caffeic acid, *p*-hydroxybenzoic, ellagic, and benzoic. Soaked fenugreek seeds (SFS) were rich in pyrogallol > caffeine > vanillic > ferulic > chlorogenic > salicylic > *p*-hydroxybenzoic > caffeic acid > benzoic > ellagic. As well as, GFS contained the following order; caffeine > oleoropin > pyrogallol > ferulic > salicylic > chlorogenic > vanillic > ellagic > *p*-hydroxybenzoic > caffeic > benzoic > 3-OH tyrosol > 4-amino benzoic > gallic. Finally, BFS was rich in pyrogallol > caffeine > vanillic > ferulic > oleoropin > chlorogenic > *p*-hydroxybenzoic > benzoic > caffeic > ellagic.

ADFL is rich in flavonoid components according to the following order; catechin > apigenin-6-*O*-arabinose-8-*O*-galactose > kaempferol-3,2-*p*-coumaroyl glucose > hesperidin > apigenin-7-*O*-glucoside. On the other side, the major flavonoid compounds in UFS were hesperidin (83.70 mg 100 g^−1^ D.W.), apigenin-6-*O*-arabinose-8-*O*-galactose (38.65 mg 100 g^−1^ D.W.), quercitrin (15.93 mg 100 g^−1^ D.W.) and catechin (14.19 mg 100 g^−1^ D.W.). Variations in the contents of flavonoid components had resulted after the household treatments as the followings; SFS were rich in hesperidin (67.45 mg 100 g^−1^ D.W.), apigenin-6-*O*-arabinose-8-*O*-galactose (40.20 mg 100 g^−1^ D.W.), catechin (12.36 mg 100 g^−1^ D.W.) and quercitrin (11.32 mg 100 g^−1^ D.W.), GFS rich in hesperidin (110.30 mg 100 g^−1^ D.W.), apigenin-6-*O*-arabinose-8-*O*-galactose (80.55 mg 100 g^−1^ D.W.), catechin (40.09 mg 100 g^−1^ D.W.), quercitrin (12.29 mg 100 g^−1^ D.W.) and rutin (10.75 mg 100 g^−1^ D.W.), and finally, BFS rich in hesperidin (102.00 mg 100 g^−1^ D.W.), apigenin-6-*O*-arabinose-8-*O*-galactose (28.17 mg 100 g^−1^ D.W.) and quercitrin (27.65 mg 100 g^−1^ D.W.).

The plant family most abundant in phytoestrogens is *Fabaceae*. The hormone-like bisphenol phytoestrogens, the isoflavonoids including daidzein and genistein, are of great interest because of their estrogenic, anti-estrogenic, anti-carcinogenic, antiviral, antifungal, and antioxidant activities^19^. In our work, four isoflavonoids (phytoestrogens) have been screened using an HPLC instrument and included daidzein, genistein, biochanin A, and isoformononetin. The data revealed that ADFL had the highest contents of isoflavonids, mainly isoformononetin and daidzein (3.87 and 3.14 mg 100 g^−1^ D.W., respectively). In contrast, UFS was high in daidzein (0.48 mg 100 g^−1^ D.W.) and genistein (0.21 mg 100 g^−1^ D.W.), and the household treatments included soaking and germination increased all contents of isoflavonids.

### Antioxidant activity of the selected samples

“The most common antioxidant methods are ABTS^·+^ and DPPH^•^. DPPH free radical (DPPH^·^) does not require any special preparation; in contrast, the ABTS radical cation (ABTS^·+^) has generated by enzymes or chemical reactions^20^”. “Another important difference is that ABTS^·+^ can dissolve in aqueous and organic media, due to their hydrophilic and lipophilic nature of the compounds in samples. On the other side, DPPH can only dissolve in organic media, especially in ethanol, this being an important limitation when interpreting the role of hydrophilic antioxidants. Both radicals show similar bi-phase kinetic reactions with many antioxidants. Although, the ferric reducing antioxidant power (FRAP) method is based on the reduction of a ferroin analogue, the Fe^3+^ complex of tripyridyltriazine Fe (TPTZ)^3+^ to the intensely blue-colored Fe^2+^ complex Fe (TPTZ)^2+^ by antioxidants in acidic medium. However, the reducing capacity does not necessarily reflect antioxidant activity, as has been suggested by Katalinic et al.^21^ and Wong et al.^22^”.

**Figure 2:**
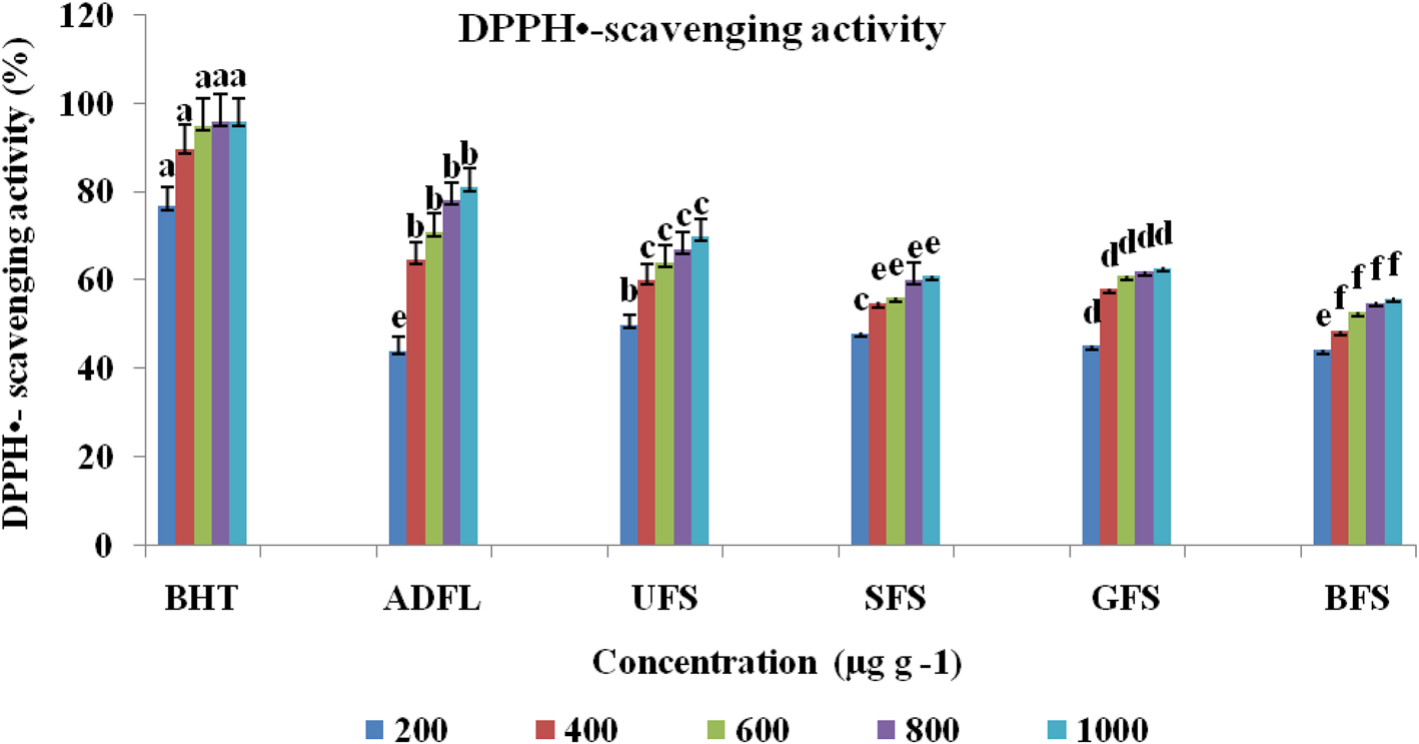
DPPH radical scavenging activity (%) of air-dried fenugreek leaves (ADFL), unprocessed (UFS), soaked (SFS), germinated (GFS) and boiled (BFS) fenugreek seeds comparing to BHT (as standard compound).

**Figure 3:**
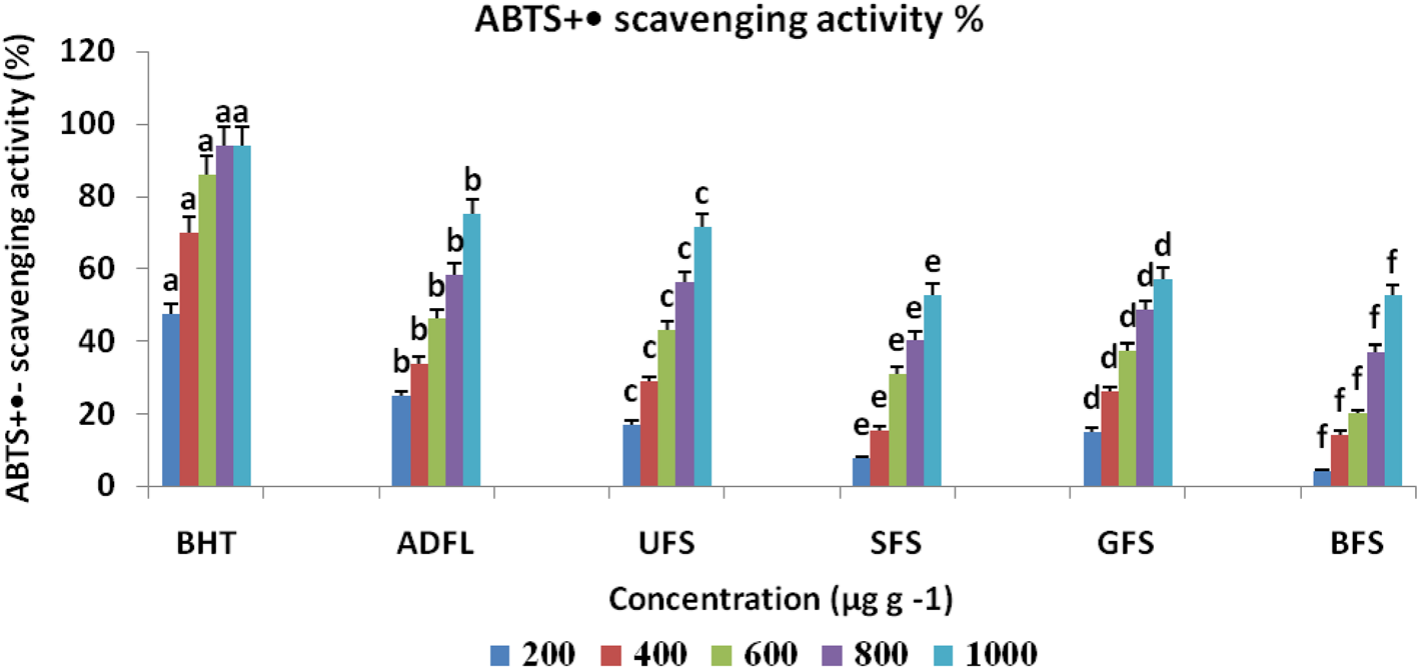
ABTS cation radical scavenging activity (%) of air-dried fenugreek leaves (ADFL), unprocessed (UFS), soaked (SFS), germinated (GFS) and boiled (BFS) fenugreek seeds comparing to BHT (as standard compound).

**Figure 4:**
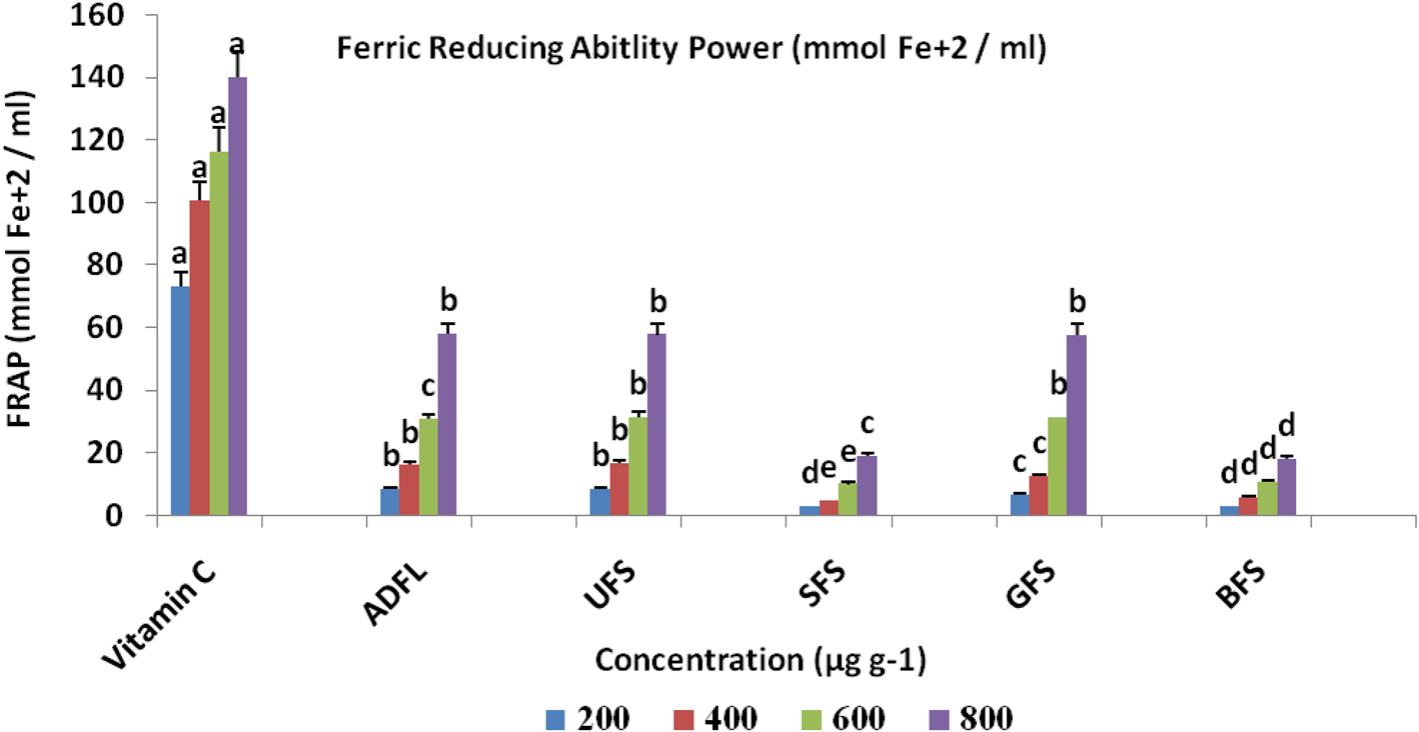
Ferric reducing antioxidant power (FRAP) (μmol Fe^2+^ ml^−1^) of air-dried fenugreek leaves (ADFL), unprocessed (UFS), soaked (SFS), germinated (GFS) and boiled (BFS) fenugreek seeds comparing to vitamin C (as standard compound).

The results of the antioxidant activity of crude extracts of each studied samples have shown in Figs. 2, 3 and 4. These results indicated that at 1000 μg ml^−1^ADFL had the maximum antioxidant activity (81.11% and 75.01%) with IC_50_ = 330 μg ml^−1^, as shown in Table 2, according to both DPPH and ABTS methods, respectively. ADFL has the maximum potential (57.88 μM) for the reduction of ferric ions into ferrous ions at 800 μg ml^−1^ according to FRAP assay. Furthermore, UFS at 1000 μg ml^−1^ exhibited a higher radical scavenging activity against DPPH and ABTS free radicals(70.04% and 71.40%) than SFS (61.00% and 52.50%), GFS (62.91% and 57.00%) and BFS (56.00% and 52.41%), respectively. According to the FRAP assay, at 800 μg ml^−1^, UFS showed more potential (57.60 μM) for ferric ions reduction than SFS (19.00 μM), GFS (57.52 μM) and BFS (18.29 μM).

**Table 2:** Antioxidant activity of crude extracts of air-dried fenugreek leaves (ADFL), unprocessed fenugreek seeds (UFS), soaked fenugreek seeds (SFS), germinated fenugreek seeds (GFS) and boiled fenugreek seeds (BFS) comparing to BHT (as standard components).

	DPPH^·^	ABTS^+·^
IC_50_ (μg ml^−1^)		
BHT	–	240.00
ADFL	330.00	638.07
UFS	200.00	697.85
SFS	300.00	950.00
GFS	276.80	847.92
BFS	480.00	950.00

According to the previous data on TP and TF, there is a linear correlation between the TP and TF and each of the free radicals (DPPH and ABTS) scavenging activities and the reduction of ferric ions into ferrous ions.

Household treatments of the fenugreek seeds reduced the in-vitro antioxidant property. These results are compatible with the earlier findings of Hooda and Jood^13^ and Shakuntala et al.^14^. In contrast, Pandey and Awasthi^15^ approved that soaking and germination enhanced the total phenolic content and the antioxidant activity of fenugreek seed flour compared to raw seeds flour.

### Antimicrobial activity

#### Antibacterial activity and MIC of the studied samples

The antibacterial activities of the alcoholic crude extracts of each studied samples were evaluated against four foodborne pathogenic Gram-positive and four foodborne pathogenic Gram-negative bacteria, as shown in Table 3. The data illustrated that crude extract of the ADFL exhibited the highest antibacterial activity against *B. cereus*, *Staph. aureus*, *Staph. scuiri* and *S. typhi* with inhibition zones 11.87, 11.61, 11.60 and 11.30 mm, respectively, compared to the positive control (tetracycline). On the other side, UFS had the maximum activity against both *S. enterica* (11.11 mm) and *S. typhi* (10.35 mm). Soaked and germinated fenugreek seeds (SFS and GFS) had the highest activity against *S. typhi* (9.52 and 9.71 mm), *S. enterica* (9.34 and 9.52 mm) and *P. aeruginosa* (9.00 and 9.15 mm), respectively. Finally, BFS had the greatest activity on *E. coli* O157 H7, *S. enteric* and *P. aeruginosa* with inhibition zones were 8.50, 8.18 and 8.12 mm, respectively.

**Table 3:** Antibacterial activity of fenugreek leaves, unprocessed and processed seeds against foodborne pathogenic bacterial strains.

Pathogenic bacteria	Inhibition zones (mm) (Mean ± STDEV*)						
	Negative control	Positive control	ADFL	Fenugreek seeds			
				UFS	SFS	GFS	BFS
(1) *B. cereus*	0	25.91 ± 2.00^b^	11.87 ± 1.01^a^	8.99 ± 0.54^cd^	7.15 ± 0.42^c^	7.41 ± 0.49^c^	7.00 ± 0.411^b^
(2) *L. monocytogenes*	0	20.66 ± 1.46^c^	10.00 ± 0.79^bc^	9.42 ± 0.61^c^	8.01 ± 0.50^b^	8.72 ± 0.51^b^	7.60 ± 0.51^b^
(3) *S. sciuri*	0	28.97 ± 2.02^a^	11.61 ± 0.10^a^	9.72 ± 0.72^c^	7.80 ± 0.51^c^	7.83 ± 0.42^c^	7.15 ± 0.42^b^
(4) *S. aureus*	0	25.86 ± 1.94^b^	11.30 ± 0.79^a^	8.83 ± 0.56^cd^	7.43 ± 0.44^c^	7.61 ± 0.50^c^	7.31 ± 0.42^b^
(5) *E. coli O157 H7*	0	10.88 ± 0.88^e^	10.09 ± 0.89^bc^	9.31 ± 0.50^c^	8.33 ± 0.51^b^	8.41 ± 0.54^b^	8.50 ± 0.50^a^
(6) *S. typhi*	0	25.06 ± 1.97^b^	11.60 ± 0.79^a^	10.35 ± 0.82^b^	9.52 ± 0.60^a^	9.71 ± 0.71^a^	7.77 ± 0.40^b^
(7) *S. enterica*	0	25.96 ± 2.02^b^	10.71 ± 0.69^a^	11.11 ± 0.79^a^	9.34 ± 0.57^a^	9.52 ± 0.63^a^	8.18 ± 0.51^a^
(8) *P. aeruginosa*	0	16.00 ± 1.94^d^	9.11 ± 0.58^c^	9.42 ± 0.75^cc^	9.00 ± 0.52^ab^	9.15 ± 0.60^a^	8.12 ± 0.50^a^

n = 3, *STDEV: Standard deviation, Different subscripts within column are significantly different at the 5% level, Negative control: DMSO, Positive control: Tetracycline.

**Figure 5:**
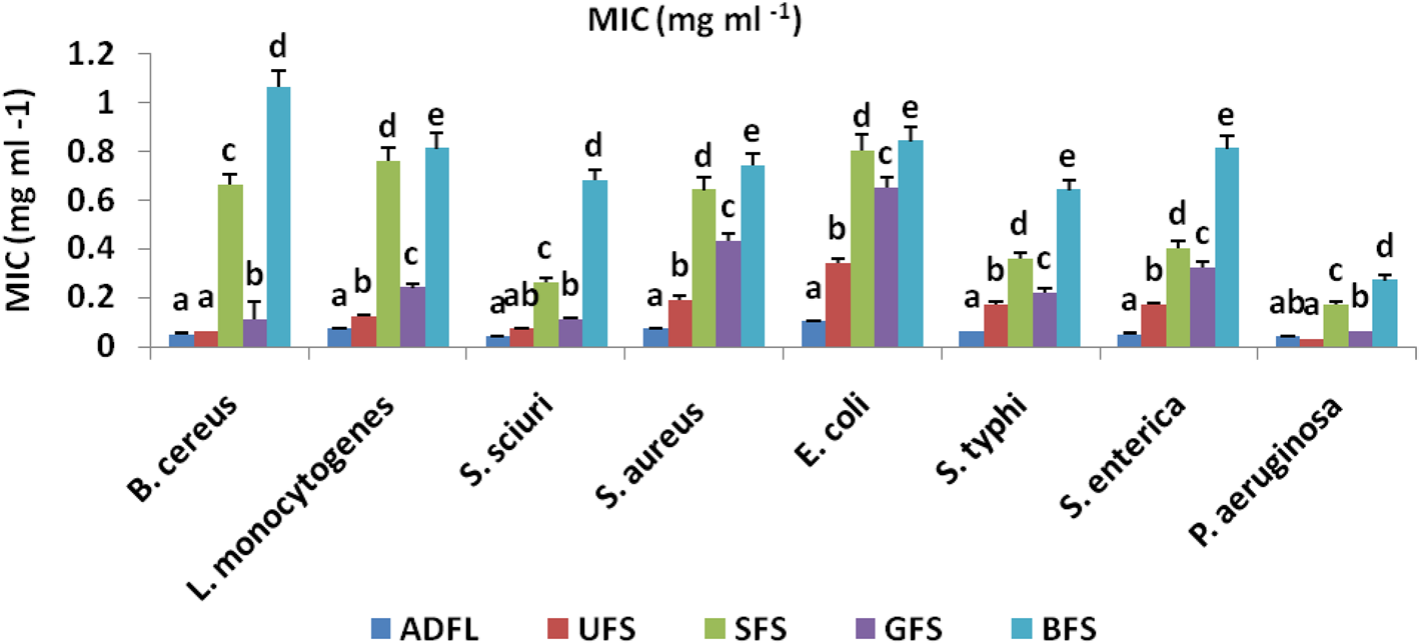
Minimum inhibitory concentrations (MIC) (mg ml^−1^) of crude extracts of air-dried fenugreek leaves (ADFL), unprocessed (UFS), soaked (SFS), germinated (GFS) and boiled (BFS) fenugreek seeds against some foodborne pathogenic bacterial strains.

As illustrated in Fig. 5, ADFL had the highest MIC values (0.04 mg ml^−1^) against each of *Staph. Scuiri* and *P. aeruginosa*, but the lowest MIC value (0.10 mg ml^−1^) was observed against *E. coli* O157 H7.On the other side, UFS had the highest MIC value (0.03 mg ml^−1^) against *P. aeruginosa* and the lowest MIC value (0.19 mg ml^−1^) against *Staph. aureus.* The maximum MIC values for each SFS, GFS, and BFS were against *P. aeruginosa* with 0.17, 0.06, and 0.27 mg ml^−1^, respectively.

#### Antifungal activity and MIC of the studied samples

Antifungal activities of the crude extracts of selected samples against eight micotoxigenic fungal strains are shown in Table 4. Data showed that ADFL had the highest activity against *A. ochraceus* and *F. proleferatum* with zones of inhibition found to be 11.01, and 10.74 mm, respectively. Also, UFS and SFS had the highest activity against *A. ochraceus* (10.75, and 8.38 mm, respectively). Germinated seeds exhibited the greater property against both of *A. ochraceus and A. purasiticus* with inhibition zones found to be 9.18 and 9.02 mm, respectively. Finally, BFS showed the highest antifungal activity against *A. carbonarius*, *A. flavus* and *P. verrucosum* with inhibition zones 8.11, 8.00, and 8.00 mm, respectively.

**Table 4:** Antifungal activity of fenugreek leaves, unprocessed and processed seeds against some mycotoxinogenic fungal strains.

Mycotoxinogenic fungi	Inhibition zones (mm) (Mean ± STDEV*)						
	Negative control	Positive control	ADFL	Fenugreek seeds			
				UFS	SFS	GFS	BFS
(1) *A. carbonarius*	0	18.78 ± 1.62^c^	10.2 ± 0.86^ab^	9.36 ± 0.52^bc^	8.00 ± 0.51^ab^	8.61 ± 0.49^a^	8.11 ± 0.50^a^
(2) *A. flavus*	0	23.33 ± 2.22^b^	8.52 ± 0.52^c^	8.50 ± 0.51^cc^	8.30 ± 0.55^a^	8.48 ± 0.51^a^	8.00 ± 0.52^ab^
(3) *A. niger*	0	23.12 ± 1.97^b^	9.24 ± 0.66^b^	8.76 ± 0.49b^c^	7.63 ± 0.43^b^	8.16 ± 0.55^a^	7.15 ± 0.51^b^
(4) *A. ochraceus*	0	19.42 ± 1.42^cc^	11.01 ± 0.97^a^	10.75 ± 0.87^a^	8.38 ± 0.53	9.02 ± 0.71^a^	8.15 ± 0.41^a^
(5) *A. purasiticus*	0	27.01 ± 2.00^a^	9.20 ± 0.56^b^	9.00 ± 0.77^bc^	8.16 ± 0.61^ab^	8.34 ± 0.62^a^	7.52 ± 0.43^ab^
(6) *A. wasterdijikia*	0	23.92 ± 1.83^ab^	10.22 ± 0.65^ab^	9.46 ± 0.71^bc^	7.37 ± 0.49^b^	8.02 ± 0.72^a^	7.46 ± 0.42^ab^
(7) *F. proleferatum*	0	14.01 ± 1.04^a^	10.74 ± 0.71^a^	9.36 ± 0.63^bc^	8.02 ± 0.62^ab^	9.18 ± 0.72^a^	7.55 ± 0.59^ab^
(8) *P. verrucosum*	0	22.99 ± 1.99^bc^	9.83 ± 0.62^ab^	9.01 ± 0.58^bc^	8.00 ± 0.66^ab^	8.91 ± 0.70^a^	8.00 ± 0.55^ab^

n = 3, *STDEV: Standard deviation, Different subscripts within column are significantly different at the 5% level, Negative control: DMSO, Positive control: Nestatin.

**Figure 6:**
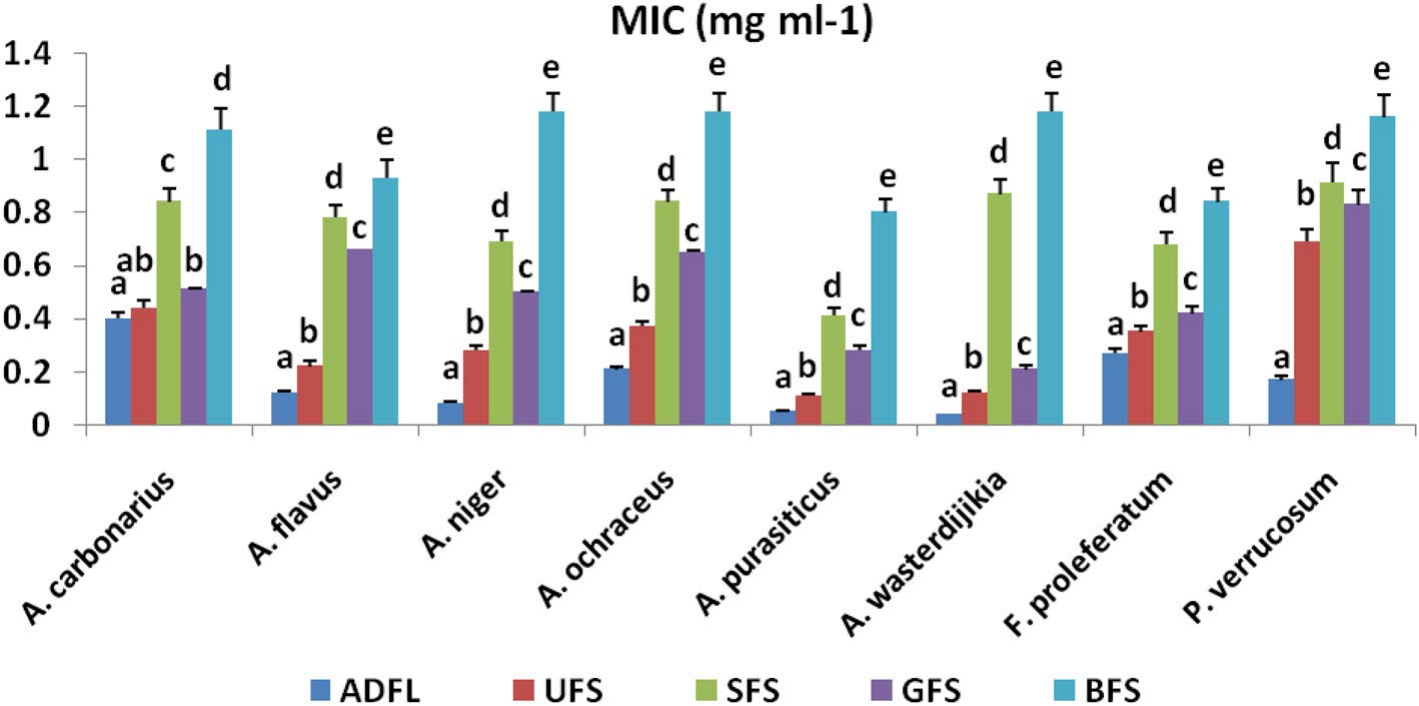
Minimum inhibitory concentrations (MIC) (mg ml^−1^) of crude extracts of air-dried fenugreek leaves (ADFL), unprocessed (UFS), soaked (SFS), germinated (GFS) and boiled (BFS) fenugreek seeds against some mycotoxinogenic fungal strains.

Figure 6 illustrates the MIC values of the tested samples against each studied fungal strain. The most effective levels of MIC values were recorded at 0.04 and 0.21 mg ml^−1^ with ADFL and GFS, respectively, against *A. wasterdijikia.* Whereas, UFS, SFS, and BFS crude extracts recorded the most significant levels of MIC values, which were 0.11, 0.41, and 0.80, respectively, against *A. purasiticus.*

Al-Abdeen^23^ studied the antibacterial activity of aqueous and some organic compound extracts of stems, leaves, seeds, and roots of fenugreek against three Gram– negative and one Gram–positive bacteria by well-diffusion and colony-assay methods. The microorganisms were *Staph.aureus, E.coli, P.aeruginosa* and *K*. spp. All plant extracts did not exhibit any inhibitory activity against any of the microorganisms tested by the well-diffusion and colony-assay techniques. Abdalah^24^ found that the extract of fenugreek seeds at concentrations of 1000, 500, and 250 mg ml^−1^ inhibited the growth of *Streptococcus pyogenes*. The methanolic extract of fenugreek seeds with concentrations of 1000, 500, 250, and 125 mg ml^−1^ inhibited the growth *Staphylococcus aureus*. The aqueous extract for the fenugreek seeds was less active then methanol extract against the growth of pathogenic bacteria. Premanath^25^ studied the antimicrobial activity of various fenugreek extracts which were screened by disc diffusion method and ethanol extract was found to be more potent. The Minimum Inhibitory Concentration (MIC) of ethanol extract determined by broth dilution method showed a MIC value of 1 mg ml^−1^ for *Staphylococcus aureus* and *Pseudomonas aeruginosa.* Nandagopal et al.^26^ evaluated the phytochemical analysis for antibacterial activity of the seed extracts of *Trigonella foenum-graecum* L. against pathogenic bacteria like Gram positive (*Staphylococcus aureus*) and Gram negative (*E. coli, Pseudomonas aeruginosa, and Klebsiella pneumonia*) bacteria by in vitro* agar well diffusion method.* The seed extracts showed more inhibitory action on *Klebsiellap neumonia and Pseudomonas aeruginosa* than *E. coli, Staphylococcus aureus.* EI Nour et al.^27^ investigated the antimicrobial activity of petroleum ether extract of seeds and callus derived from hypocotyls and cotyledons explants of fenugreek. The petroleum ether extract showed highest antimicrobial activity. Antibacterial activity of petroleum ether extract was recorded 17 ± 0.33 mm and 15 ± 0.57 mm of inhibition zone against *E. coli* and *Staphylococcus aureus*, respectively, at concentration of 250 mg ml^−1^. It showed antifungal activity against *Aspergillus niger* and *Candida albicans* with maximum zone of inhibition (20 ± 0.88 mm) against *A. niger* by conc. 250 mg ml^−1^ and (17 ± 0.57 mm) of inhibition zone against *Candida albicans* by concentration of 250 mg ml^−1^.

#### In-vitro anti-inflammatory activity of the selected samples

Industrial steroidal and non-steroidal anti-inflammatory drugs have a wide range of side effects. So, diverse works are interested in finding anti-inflammatory agents from natural sources^28^.

Our work is the first study reports the impact of household techniques on the cytotoxic activity against RAW 264.7 macrophage cell line and the anti-inflammatory property by NO assay of both fenugreek leaves and seeds crude extracts.

#### Cell cytotoxicity assay

Results presented in Table 5 illustrated that ADFL crude extract exhibited the highest cytotoxic activity (89.03%) on the RAW 264.7 cell line at a concentration of 100 µg g^−1^. Furthermore, UFS at a concentration of 100 µg g^−1^ had higher activity (73.21%) than the processed samples, SFS, GFS and BFS, which showed 38.66%, 63.00%, and 18.19% cytotoxic activity, respectively, at the same concentration. So, the household treatments declined the cytotoxic property of the fenugreek seeds against the macrophage cell line but were not affected by the fenugreek leaves. These may be due to a decrease in TP, TF, and AOX activity after each soaking, germinating and boiling process.

**Table 5:** Cytotoxicity of crude extracts of the studied samples against RAW 264.7 macrophage cell line.

	10	20	40	80	100	IC_50_ (µg g^−1^)
Cytotoxicity of RAW cells (%) (Mean ± STDEV*)						
Concentration (µg g^−1^)						
ADFL	15.21 ± 1.12^c^	30.00 ± 2.00^c^	52.23 ± 3.32^a^	73.12 ± 4.23^a^	89.03 ± 5.71^a^	37.48
UFS	29.03 ± 2.00^a^	38.11 ± 2.12^a^	45.00 ± 3.01^b^	65.02 ± 4.11^b^	73.21 ± 4.61^b^	60.16
SFS	10.01 ± 0.96^d^	15.11 ± 1.21^d^	20.03 ± 1.72^d^	33.12 ± 2.16^d^	38.66 ± 2.37^d^	–
GFS	25.22 ± 1.79^b^	31.21 ± 2.21^b^	40.21 ± 2.66^c^	52.93 ± 3.74^c^	63.00 ± 4.21^c^	71.05
BFS	8.37 ± 0.67^e^	9.00 ± 0.86^e^	13.21 ± 1.01^e^	15.91 ± 1.03^e^	18.19 ± 1.31^e^	–

*Each value represents the mean ± standard deviation *The same letter of denoting over values proves that they are not at significantly different at (*p* ≤ 0.05), and comparison is done according to treatments. *(–) means unspecified IC _50_(µg g^−1^).

#### Nitric oxide (NO) inhibition assay

Data shown in Table 6 demonstrated that ADFL at 100 µg g^−1^ exhibited the maximum inhibition (76.11%) of NO molecules. On the other side, at 100 µg g^−1^, UFS showed higher inhibition (62.11%) than each of SFS (39.91%), GFS (56.12%), and finally, BFS (33.11%).

**Table 6:** Anti-inflammatory activity of crude extracts of selected samples against nitric oxide (NO).

	10	20	40	80	100	IC_50_ (µg g^−1^)
Inhibition of NO (%) (Mean ± STDEV*)						
Concentration (µg g^−1^)						
ADFL	20.47 ± 1.67^a^	30.51 ± 1.78^a^	38.26 ± 2.62^a^	68.92 ± 4.16^a^	76.11 ± 4.49^a^	59.79
UFS	15.00 ± 1.13^b^	26.12 ± 1.92^b^	28.26 ± 2.01^b^	59.21 ± 3.76^b^	62.11 ± 3.91^b^	72.55
SFS	8.02 ± 0.51^d^	13.01 ± 1.01^d^	21.00 ± 1.41^d^	31.01 ± 2.11^d^	39.91 ± 2.11^d^	–
GFS	13.11 ± 1.00^c^	20.21 ± 1.88^c^	25.11 ± 1.93^c^	50.31 ± 3.41^c^	56.12 ± 3.16^c^	82.77
BFS	6.12 ± 0.42^e^	7.51 ± 0.52^e^	15.73 ± 1.02^e^	26.22 ± 1.96^e^	33.11 ± 2.01^e^	–

*Each value represents the mean ± Standard deviation **The same letter of denoting over values proves that they are not at significantly different at (*p* ≤ 0.05), and comparison is done according to treatments. *(–) means unspecified IC _50_ (µg g^−1^).

## Materials and methods

### Chemicals

Ethanol (95%), methanol (96%), dimethyl sulfoxide (DMSO), sodium carbonate, aluminum chloride, sodium nitrate(III), acetic acids, potassium persulphate, sodium hydroxide, butylated hydroxytoluene (BHT), ascorbic acid were purchased from El Gomhoryia, El Nasr and Middle East Pharmaceutical Chemical companies, Egypt and the solvents were purified before using.

The Folin–Ciocalteau reagent, 2,2-diphenyl-1-picrylhydrazyl (DPPH), 2,2′-azino-bis(3-ethylbenzothiazoline-6-sulfonic acid diammonium salt) (ABTS), 2,4,6-tri(2-pirydyl-s-triazine (TPTZ) were purchased from Sigma-Aldrich Chemie (Steinheim, Germany). Chemicals, solvents, all standards of phenolic acids, flavonoids and isoflavonoids used for fractionation and identification by HPLC were purchased from Sigma-Aldrich Chemie (Steinheim, Germany).

### Plant materials

Our used of plant material and all methods in our research complies with all applicable local, regional, national, and international regulations.

#### Air-dried fenugreek leaves (ADFL)

Fresh and healthy leaves of fenugreek (*Trigonella foenum-graecum* Linn.) were purchased from the local market of Egypt in December 2019 and identified by the Faculty of Science, Cairo University. The leaves have washed thoroughly with tap water, and the surface water has removed by air-drying under shade for 15 days. The leaves have subsequently dried in a hot air-oven at 50 °C for 4 h, homogenized to a fine powder and then stored at 4 °C.

#### Fenugreek seeds

Two kilograms from fenugreek seeds were also purchased from the local market of Egypt, identified by the Faculty of Science, Cairo University, and divided into four groups, as the followings;

*1st group (Untreated fenugreek seeds, UFS)*, 500 g of seeds were manually cleaned to remove dust and foreign particles, crushed into a fine powdered flour with the help of Moulinex blender LM 241 and were sieved in a 0.5 mm mesh size. The powdered flour has been kept at 4 °C to prevent changes till further analyses.

*2nd group (Soaked fenugreek seeds, SFS)*, 500 g of raw seeds were soaked in tap water at the ratio of 1:5 (w/v) for 12 h at room temperature. After pouring off the soaking water, seeds were air-dried in shade for 5 days followed by hot air-oven drying at 50 °C for 4 h in a conventional oven, and stored at 4 °C.

*3rd group (Germinated fenugreek seeds, GFS)*, 500 g of raw seeds were soaked in tap water at the ratio of 1:5 (w/v) for 12 h at room temperature. After pouring off the soaking water, seeds were kept in the dark for germination (tied in cotton cloth) at 20 °C for 60 h in darkness. After harvesting the sprouts, they were air-dried for 5 days followed by hot air-oven drying at 50 °C for 4 h in a conventional oven, and stored at 4 °C.

*4thgroup (Boiled fenugreek seeds, BFS)*, 500 g of raw seeds were put in 2 L beaker containing 1250 ml tap H_2_O (1:5 w/v). The sample was boiled on a hot plate for 10 min and then, after water was discarded, the boiled seeds were air-dried in shade for 5 days followed by hot air-oven drying at 50 °C for 4 h in a conventional oven, and stored at 4 °C.

#### Extraction procedure of samples

One hundred grams of each sample were extracted with 1 L (1:10 w/v) 80% ethanol in distilled H_2_O by sonication for 60 min. Extraction had repeated three times. After filtration, each extract condensed to dryness (resulting in crude extracts) using a rotary evaporator at 40 °C. The obtained residue has collected to calculate the yield and finally stored in the freezer for further biochemical and in-vitro biological analyses.

A known weight of each crude extract obtained from rotary evaporation has dissolved in each ethanol 80% for determination of TP, TF, DPPH^·^, ABTS^+·^, and FRAB^·^, and in dimethyl sulphoxide (DMSO) for both *in-vitro* antimicrobial and anti-inflammatory activities.

Recovery of the extract was calculated as yield (%) using the following equation:

$${\text{Yield }}\left( \% \right) = [{\text{W}}_{{\mathrm{f}}} / {\text{W}}_{{\mathrm{i}}} ] \times {1}00,$$

where W_f_ is the final weight of the crude extract and W_i_ is the initial weight of the tested sample.

### Determination of total phenolic content (TP)

Total phenolic content of ethanol extracts was evaluated according to Singleton and Rossi^29^, using of Folin–Ciocalteau reagent. Total phenolic content was expressed as mg of gallic acid equivalents (GAE) per g dr weight (D.W.) of sample. All determinations were performed in triplicates.

### Determination of total flavonoid content (TF)

Total flavonoid content was analyzed by a spectrophotometric method described by Boateng et al.^30^. Total flavonoid content was expressed as mg of quercetin equivalents (QE) per g D.W. of sample. All determinations were performed in triplicates.

### Fractionation of phenolic and flavonoid components by HPLC

A high-performance liquid chromatography system equipped with a variable wave length detector (Agilant technologies, Germany) 1200 series. Also the HPLC was equipped with auto-sampler, Quaternary pump degasser and column compartment set at 35 °C. Analyses were performed on a C18 reverse phase (BDS 5 μm, Labio, Czech Republic) packed stainless-steel column (4 × 250 mm, i.d.).To determine phenolic and flavonoids compounds, samples were prepared according to the method described by^31^. All chromatograms were plotted at 280 nm to estimated phenolic compounds and at 330 nm for flavonoids and isoflavonoids. All components were identified and quantified by comparison of peak areas with external standards**.**

### Antioxidant activity of alcoholic extracts of fenugreek samples

#### Determination of DPPH^•^ scavenging activity

In order to determine DPPH radical-scavenging activity, a method described by Moure et al.^32^ was used with minor modification. Various concentrations of each sample (200, 400, 600, 800 and 1000 µg g^−1^) were prepared from the stock solution (10 mg ml^−1^). The DPPH radical-scavenging activity in the extracts was expressed as percentage inhibition activity.

The percentage inhibition activity was calculated from [(A_C_ − A_S_)/A_C_] × 100.

A_C_ is the absorbance of the control, and A_S_ is the absorbance of the sample. The analyses were carried out in triplicate.

#### Determination of ABTS^•+^ scavenging activity

The free radical-scavenging activity has been determined by the ABTS radical cation decolorization assay described in^33^. The results were expressed as the percentage inhibition activity which calculated from [(A_c_ − A_s_)/A_c_] × 100. A_c_ is the absorbance of the control, and A_s_ is the absorbance of the sample. All determinations have been performed in triplicate.

#### Determination of ferric reducing antioxidant power (FRAP)

The activity of the extracts for reducing the ferric ion was assayed according to the method of Benzie and Strain^34^ method. Ferric reducing antioxidant power was expressed as μmol of Fe^2+^ per 100 g D.W. of each sample.

### In-vitro antimicrobial activity

#### Tested microorganisms

The antimicrobial activity of alcoholic extracts of fenugreek leaves, unprocessed and processed seeds was evaluated against eight foodborne pathogenic bacterial strains and eight pathogenic fungal species. Four Gram-positive bacterial strains were *Bacillus cereus* (EMCC 1080), *Listeria monocytogenes* (ATCC 7644), *Staphylococcus sciuri* (2–6) and *Staphylococcus aureus* (ATCC 13565), and four Gram-negative bacterial strains *Escherichia coli* O157 H7 (ATCC 51659), *Salmonella typhi* (ATCC 25566), *Salmonella entrica* (SA 19992307) and *Pseudomonas aeruginosa* (NRRL B-272). All the studied bacterial strains were grown on nutrient agar slants at 37 °C for 24 h and then kept at 4 °C till use.

The tested fungal strains included *Aspergillus carbonarius* (ITAL 204), *Aspergillus flavus* (NRRL 3357), *Aspergillus niger* (IMI 288550), *Aspergillus ochraceus* (ITAL 14), *Aspergillus parasiticus* (SSWT 2999), *Aspergillus westerdijikia* (CCT 6795), *Fusarium proliferation* (MPVP 328) and *Penicillium verrucosum* (BFE 500) were grown on potato dextrose agar slants (PDA) at 25 °C for 5 days and then kept at 4 °C till use.

#### Agar disc-diffusion assay

Agar disc-diffusion assay was used for evaluation the antimicrobial activity of alcoholic extracts against some foodborne pathogenic bacteria and fungi according to Kavanagh^35^ method. The plates were incubated for 24 h at 37 °C and after the incubation period, the diameters of the cleared zones of inhibition (millimeter) were measured. Dimethyl sulphoxide (DMSO) was used as the negative control, tetracycline used as positive control. Mean and standard deviation (STDEV) values were tabulated.

#### Measurement of minimum inhibitory concentration (MIC)

MIC against fungi was studied by using the technique of Perrucci et al.^36^. The prepared plates were centrally inoculated with 3 μl of fungal suspension (10^8^ CFU ml^−1^; 0.5 McFarland standards). The plates were incubated at 25 °C for 24–48 h. At the end of the incubation period, mycelial growth was noticed and MIC was determined.

### In-vitro anti-inflammatory activity

#### Cell cultivation

RAW 264.7 macrophage cell line was acquired from the ATCC (American type culture collection). Cells were cultivated in RPMI, 1640 medium (Institute of Roswell Park Memorial), and subjoined with 1% pen/strep and 10% heat-inoperative fetal bovine serum. Cells were transferred in a moistened incubator, in an ambient of 5% CO_2_ at 37 °C, and they were subculture two times before the assay.

#### Proceedings

The following proceedings were obtained in a sterile area using a laminar bio safety flow cabinet class II level (Baker, SG403INT, Sanford, ME, USA). RAW 264.7 cells were suspended in RPMI medium. After 24 h of seeding 1 × 10^5^ cells per well (in 96—well plates) and incubated for one day for the assay. Cells were then processed with the specimens at different concentrations of 10, 20, 40, 80 and 100 µg g^−1^ and incubated for 60 min. Cells were then enhanced with 10 μg ml^−1^ of LPS, as a negative control, for another one day. The supernatant was transferred wisely to a new 96-well plate and processed for NO determination, while the cells stayed in the old plate were used for the MTT protocol, to determine the percentage of the viable cells. Specimens (stock) were dissolute in DMSO, and the working specimens were prepared in the media. Viable cells were determined by the reduction of mitochondrial dependence of yellow MTT (3-(4,5-dimethylthiazol-2-yl)-2,5-diphenyltetrazolium bromide) to purple formazan^37^. The percent of change in the number of viable cells was measured according to the following equation:

$$\left[ {\left( {{\text{R}}*/{\text{R}}^{0} } \right) \, - { 1}} \right] \times { 1}00$$

As R* is reading of the extract, and R^0^ reading of the control.

#### Nitric oxide (NO) protocol

The production of nitric oxide was processed by determining nitrite in the supernatants of cultivated RAW 264.7 macrophages. The protocol was processed with slight modification as previously described ^38^. After pre-incubation for one day of RAW 264.7 cells (1 μg ml^−1^) with LPS (10 μg ml^−1^), the quantity of nitrite, which is considered a stable NO—metabolite, and used as an indicator of NO—production in the culture medium, it was estimated using a reagent of (0.1% naphthyl ethylene diamine dihydrochloride + 1% sulfanil amide + 2.5% phosphoric acid), and this reagent is commonly known as Griess reagent. A 50 μl volume of the Griess reagent was mixed with 50 μl of the cell culture medium. Afterward, the mixture was incubated at ambient temperature for 15 min, and the absorbance was measured using a microplate multi-well reader (Model 3350, Hercules, California, USA, Bio-Rad Laboratories Inc.) at 540 nm. In every single experiment, a new culture medium was used as a blank. The amount of nitrite was estimated from a sodium nitrite standard curves phrased in the following equation:

$${\text{Inhibition }}\left( \% \right){\text{ of nitric oxide}} = \left[ {\left( {{\text{Control}} - {\text{Test}}} \right)/{\text{Control}}} \right] \times {1}00$$

### Statistical analysis

All assays used in this work were evaluated triple times, and the data obtained were represented by the mean ± standard deviation (STDEV). Statistical Analysis Software (SAS 9.1) was applied for the statistical analysis of data, and IC_50_ was calculated by using of Graphed prisms. One-way analysis of variance (ANOVA) was used to analyze the difference between groups by applying the least significant difference (LSD) test with 1% and 5% levels of significance (*p* < 0.05).

## Conclusions

Fenugreek seeds have a bitter taste due to saponins and specific smell due to alkaloids and volatile oils, which limit their useand acceptability in the food industry. It has been possible debittered by using various household treatments such as soaking, germinating, boiling, etc. Among all the studied samples, ADFL exhibited the highest in-vitro antioxidant, antibacterial, antifungal, and anti-inflammatory properties due to their high contents of phenolics, flavonoids, and isoflavonoids. Each soaking, germinating, and boiling treatments lowered the in-vitro biological activities because a decreasing occurred in TP, TF, and AOX activities. Finally, air-dried fenugreek leaves and unprocessed seeds must use in both industrial and pharmaceutical fields as an excellent natural antioxidant, antibacterial, antifungal and anti-inflammatory agents, as well as a natural source of potential phenolics, flavonoids, isoflavonoids, steroidal saponins, alkaloids, etc. in medicinal field.

## Data Availability

Samples of the compounds and data used during the current study are available from the corresponding author.

## Abbreviations

ABTS: 2,2-Azinobis (3-ethylbenothiazoline-6-sulfonic acid)
AC: Absorbance of control
ADFL: Air-dried fenugreek leaves
As: Absorbance of sample
ATCC: American type culture collection
BFS: Boiled fenugreek seeds
BHT: Butylated hydroxytoluene
CFU: Colony forming unit
CNS: Central nervous system
COX-2: Cyclooxygenase-2
D.W.: Dry weight
DMSO: Dimethyl sulphoxide
DPPH: 2,2-Diphenyl-1-picrylhydrazil
EMCC: Egypt microbial culture collection
FRAP: Ferric reducing antioxidant power
GAE: Gallic acid equivalents
GFS: Germinated fenugreek seeds
HPLC: High performance liquid chromatography
i–NOS: Inducible nitric oxide synthase
LPS: Lipopolysaccharide
LSD: Least significant difference
MAPK: Mitogen-activated protein kinase
MIC: Minimum inhibitory concentration
NF-κB: Nuclear factor kappa light chain enhancer of activated B cells
NO: Nitric oxide
NRRL: Northern Regional Research Laboratory
PDA: Potato dextrose agar
PGE2: Prostaglandin E2
QE: Quercetin equivalents
ROS: Reactive oxygen species
STDEV: Standard deviation
SFS: Soaked fenugreek seeds
TF: Total flavonoid
TP: Total phenolic
UFS: Unprocessed fenugreek seeds
